# Dyspnea and COVID-19: A Review of Confounding Diagnoses during the Postpartum Period

**DOI:** 10.1055/s-0041-1736304

**Published:** 2021-12-06

**Authors:** Clara Nunes Castro, Pedro Paulo Machado Lopes, Jussara Mayrink

**Affiliations:** 1 Department of Gynecology and Obstetrics, Universidade Federal de Minas Gerais, Belo Horizonte, MG, Brazil

**Keywords:** dyspnea, postpartum period, puerperium, COVID-19 diagnosis, dispneia, pós-parto, puerpério, diagnóstico de COVID-19

## Abstract

The puerperium is a complex period that begins with placental delivery and lasts for 6 weeks, during which readaptation of the female organism and redistribution of blood volume occur. This period is conducive to the occurrence of thromboembolic events. In the context of the SARS-CoV-2 pandemic, the virus responsible for COVID-19, the attention of the scientific community and health professionals has been focused on obtaining insights on different aspects of this disease, including etiology, transmission, diagnosis, and treatment. Regarding the pregnancy–postpartum cycle, it is opportune to review the clinical conditions that can occur during this period and to investigate dyspnea as a postpartum symptom in order to avoid its immediate association with COVID-19 without further investigation, which can lead to overlooking the diagnosis of other important and occasionally fatal conditions.

## Resumo

O puerpério é um período complexo que se inicia com a dequitação placentária e dura por 6 semanas, no qual a readaptação do organismo materno e a redistribuição do volume sanguíneo ocorrem, além de ser também um cenário propício para eventos pró-trombóticos. No contexto da pandemia de SARS-CoV-2, vírus responsável pela COVID-19, a atenção da comunidade científica e dos profissionais da saúde está voltada a elucidar os aspectos da doença, como a etiologia, a transmissão, o diagnóstico e o tratamento. Considerando o ciclo gravídico-puerperal, é oportuna a revisão de condições clínicas que ocorrem durante este período e que apresentam a dispneia como sintoma, a fim de evitar que ela seja automaticamente associada à COVID-19 sem investigações aprofundadas, o que pode levar à negligência do diagnóstico de outras condições importantes e que podem ser, por vezes, fatais.

## Introduction

The puerperium is the period that starts with placental delivery and lasts for 6 weeks. It is a complex phase during which women face numerous challenges related to physiological changes associated with the end of pregnancy, intensified by psychological changes related to their new life as a mother. During the puerperium, a woman may visit the emergency department for many reasons, including dyspnea, which is a cardinal symptom that always poses a challenge owing to the broad spectrum of differential diagnoses, ranging from psychiatric disorders to pulmonary and cardiovascular diseases. 1

According to Campbell, 2 dyspnea is a subjective experience of respiratory discomfort that can only be known through the report of a patient. The author described that treating dyspnea begins with managing the underlying condition or recognizing the provoking factors. Pregnancy and the postpartum period predispose women to respiratory infections, and these women are more likely to develop severe illness after infection with respiratory viruses. 3 During the pandemic caused by severe acute respiratory syndrome coronavirus 2 (SARS-CoV-2), the virus responsible for coronavirus disease 2019 (COVID-19), emergency departments have been visited by patients presenting with dyspnea. As dyspnea is also one of the symptoms of COVID-19, clinicians should be aware of all possible diagnoses associated with this symptom. 4 5

The puerperium is also a time during which women are predisposed to the development of postpartum psychiatric disorders, which can include depression, anxiety, and postpartum psychosis (a more severe manifestation), all of which can present dyspnea as the primary symptom. 6 Moreover, during the postpartum period, a procoagulation biochemical scenario occurs that, in addition to changes in the anatomy and cardiovascular system of the woman, facilitates the development of conditions such as pulmonary thromboembolism and peripartum cardiomyopathy (PPCM), both of which can also present dyspnea as the primary symptom. 7 8

Apart from psychiatric diagnoses, which are beyond the scope of the present review, our goal is to summarize and draw attention to important and possibly life-threatening conditions that fall under the umbrella of dyspnea. COVID-19 is one of many diseases that can present this symptom, and any physician who sees postpartum women should be aware of these possibilities to provide an intervention as early as possible.

## Pulmonary Thromboembolism

The pregnancy–puerperal cycle is characterized by physiological changes that can facilitate the occurrence of thromboembolic events, mostly venous thromboembolism (VTE), by increasing coagulation and venous stasis. Hypercoagulability occurs due to a greater synthesis of procoagulant factors, which are important for effective hemostasis in childbirth. Meanwhile, venous stasis results from reduced venous return and compression of blood vessels, such as the inferior vena cava and the pelvic vessels, caused by increased uterine volume. The combination of these factors is responsible for the increased risk of thromboembolic events in this population, which is four to five times higher than in nonpregnant women. 8 Pulmonary embolism (PE) is the leading direct cause of death during pregnancy and in the postpartum period. 9 Approximately 1 in between 1,000 and 3,000 pregnancies is complicated by PE. 10

### Clinical Presentation and Diagnosis

Pulmonary embolism can present with nonspecific symptoms or can even be asymptomatic. Leg pain, leg discoloration, and unilateral leg swelling may appear as manifestations of deep vein thrombosis, which is a probable cause of embolism. 11 However, the most common presentations include sudden-onset dyspnea, syncope, chest pain, and hemoptysis. 12 The presence of any combination of these symptoms, especially in pregnant or postpartum women, should call attention to the possibility of PE.

The usual diagnostic evaluation for suspected cases in nonpregnant women involves the sequential application of clinical decision rules and a D-dimer test. 9 However, during pregnancy or during postpartum, the use of D-dimer is not recommended, 13 as explained below. The applicability of scores to assess PE probability is also discussed below.

The evaluation of women with suspicion of PE and leg symptoms should be made with a compression Doppler ultrasonography (CUS), since it is noninvasive and can be rapidly performed. 7 8 The association of symptoms suggesting PE and a positive CUS demands no further imaging before initiating treatment. 7

However, for women without leg symptoms, hemodynamically unstable or critically ill, chest imaging should be prioritized. It should be initiated with a chest x-ray (CXR) and, depending on its results, followed by computed tomography pulmonary angiography (CTPa) or a ventilation/perfusion scintigraphy (V/Q). 7

## D-dimer

Plasma D-dimer, a fibrin derivative produced by the degradation of fibrin by plasmin, has demonstrated a high negative predictive value for VTE in the nonpregnant population with low or moderate clinical suspicion, and may assist in screening individuals for further investigation with ultrasound or V/Q. However, in pregnant and postpartum women, its precision remains to be studied, as D-dimer levels are naturally elevated in this population and the conventional negative predictive threshold of 0.5 mg/L is associated with high rates of false-positive results during pregnancy and the postpartum period. 13 The DiPEP study reinforced this finding, showing that using D-dimer as a diagnostic marker would miss a substantial proportion of cases with PE during pregnancy and the postpartum period. 9

## Wells Modified Score and Revised Geneva Score: are they Useful for Postpartum Period?

In the nonpregnant population, the modified Wells score and the revised Geneva scores are currently used and validated for PE diagnosis as accurate clinical protocols that triage patients into low-, medium- and high-risk groups, reducing the rate of unnecessary imaging studies. 14 In a study focusing on 103 pregnant women, the negative predictive value was 100% with a Wells score < 6. However, the studied population did not include early postpartum women. 15 A retrospective study, involving early postpartum patients, applied both pretest Well and Geneva scores, and analyzed the accuracy of them to predict pulmonary embolism cases. The results were modest for both: 40.7 and 62.9% of sensitivity values and 79.4 and 81.8% negative predictive value, respectively. 14 However, these scores depend on variables that do not usually concern the peripartum period, such as “age > 65 years old”, “surgery (under general anesthesia)” or “malignant condition treatment”. Thus, apparently, the algorithms used to diagnose PE in postpartum women usually start with an imaging modality. This is also the recommendation of the Royal College of Obstetricians and Gynaecologists and of the American Thoracic Society. 16 17

## Computed Tomography Pulmonary Angiography

Computed tomography pulmonary angiography is considered the gold standard imaging test for the diagnosis of acute PE events. Its use during pregnancy is controversial, due to the requirement for contrast and radiation, even though the degree of fetal exposure is low. 8 However, during postpartum, this concern is no longer necessary. Therefore, CTPa is an important exam in postpartum women, especially in patients with abnormal CXR, in who its accuracy is higher. 7 8 It can also suggest alternative diagnoses in the absence of PE. 7

When the CTPa shows no filling defects in any branches of the pulmonary artery, the exam is considered negative and other causes must be investigated. On the other hand, the presence of these defects should be considered a positive exam, 7 and treatment should be established as soon as possible.

Computed tomography pulmonary angiography should also be recommended when V/Q is not available for further evaluation. 7

## Ventilation/Perfusion Scintigraphy

The levels of radiation on V/Q are similar to those in CTPa. Although the latter has lower fetal radiation exposure, the former has lower maternal radiation exposure, especially to the breasts. 8 Therefore, its use during pregnancy is also debatable. In puerperium, however, it should be used in women in who the suspicion for PE continues even after a negative CUS and/or a normal CXR. 7

The results of V/Q can be: (1) normal; (2) low probability; (3) intermediate probability; (4) mismatched perfusion defect. In the first one, PE is excluded and other causes of dyspnea should be investigated. The second and third results should be evaluated considering the level of clinical suspicion. If it is low, serial CUS is recommended. If it is high, a CTPa should be performed to investigate further. However, if the results of the V/Q shows mismatched perfusion defect, the diagnosis of PE is made. 7

Even though this exam has high accuracy and it is considered safe during postpartum, it is available only in high complexity centers. Therefore, in its absence, CTPa, as said before, is an adequate substitute. 7

### Prognosis and Prophylaxis

The best prophylactic measure against thromboembolism during the postpartum period is to avoid cesarean delivery, if possible. A cesarean section almost quadruples the risk of VTE compared with vaginal delivery. Anticoagulation should be considered in patients with additional risk factors for thromboembolism, although this recommendation should be individualized. Nevertheless, all women undergoing cesarean delivery should receive pneumatic compression devices, placed before the delivery and maintained until the patient is ambulatory. In addition, early mobilization after the procedure should be reinforced. 8

## Peripartum Cardiomyopathy

Peripartum cardiomyopathy is a rare, idiopathic heart disease that presents in late pregnancy or, more commonly, during the early postpartum period, appearing *de novo* at between 1 and 20 weeks postpartum. It is diagnosed when no other causes of heart failure are found and left ventricular dysfunction is detected by echocardiography, 18 19 meaning that it is a diagnosis of exclusion. Black women are at a high risk of PPCM, as are women with advanced maternal age, pre-eclampsia, multiple gestation pregnancy, and extensive use of assisted reproductive technology, which may explain its increased prevalence over the last few decades. 18 Cardiovascular risk factors such as obesity, arterial hypertension, and diabetes can also contribute to an increased prevalence of PPCM. 20 The prevalence ranges from 1 in 100 pregnancies in Nigeria (a “hot spot”) to 1 in 1,500 in the United States. It is important to emphasize that milder forms of PPCM may go undiagnosed due to their nonspecific symptoms and the low awareness of this condition. 19

### Etiology

The etiology of PPCM seems to be related to genetic predisposition, with variants in genes that encode sarcomeric proteins. 21 22 Another hypothesis relates to prolactin, a protein secreted by the anterior pituitary, which seems to be involved in cardiotoxic effects, leading to cardiomyocyte death and myocardial endothelial cell apoptosis. 18 Placental angiogenic factors, such as the soluble fms-like tyrosine kinase-1 (sFlt-1) receptor, are also correlated with this disease. The sFlt-1 receptor is an antiangiogenic protein secreted by the placenta, and its level exponentially increases toward the end of pregnancy. The remarkably high level of this endothelial factor in PPCM cases may be explained by the high prevalence of preeclampsia in this patient group.

### Clinical Presentation and Diagnosis

Dyspnea on exertion, orthopnea, paroxysmal nocturnal dyspnea, and edema of the lower extremities are some of the symptoms attributed to congestive heart failure. Chest pain can be severe and, in this case, the clinical picture may suggest a PE. 18 Although less common, cardiogenic shock that requires inotropic or mechanical circulatory support can be seen in more severe cases. 20

Echocardiography is the most useful imaging modality for PPCM. Hibbard et al. proposed a more stringent definition for diagnosing PPCM using echocardiographic criteria, as follows: left ventricular dysfunction with left ventricular ejection fraction (LVEF) < 45% and/or fractional shortening < 30% and end-diastolic dimension > 2.7 cm/m ^2^ . 23 Of these, left ventricular dysfunction with LVEF < 45% is the most reproducible and widely used criterion. 23 This finding, in the absence of an alternative explanation, strongly indicates the possibility of a PPCM diagnosis. Magnetic resonance imaging (MRI) can be used when echocardiography is technically limited. Chest radiography shows an enlarged cardiac silhouette with varying degrees of pulmonary congestion and edema. 18 Endomyocardial biopsy is not indicated.

### Prognosis

Most patients recover their LVEF, at least partially, at 6 months; however, the recovery and mortality rates vary among different epidemiological and geographic populations. In the United States, only 35% of patients with PPCM show an ejection fraction < 45%. 18 Left ventricular size and ejection fraction at the time of diagnosis are both strong predictors of left ventricular recovery. According to a cohort study, LVEF < 30% and left ventricular end-diastolic diameter > 6 cm are indicative of a decreased likelihood of left ventricular recovery, an increased need for mechanical support or transplant, and an increased risk of death. 24

## COVID-19

### Etiology

COVID-19 is an infectious disease caused by a viral agent from the coronavirus family, called SARS-CoV-2, named in reference to a similar virus (SARS-CoV) that caused an outbreak in 2002. It was first identified in China in December 2019 and was declared a pandemic by the World Health Organization (WHO) in March 2020. It primarily affects the respiratory system, but can also cause cardiovascular, gastric, and neurological complications. 25 Because of its recent appearance, information about its virulence and impact on the human body is still being collected.

The large potential for the spread of SARS-CoV-2 is impressive, as is its basic reproduction number (a parameter used to estimate the average number of secondary cases generated by an infectious case in a fully susceptible population during the early phase of an outbreak), which ranges from 1.4 to 6.49. 26

The incubation period varies from 2 to 15 days, and the mode of human-to-human transmission is mainly via droplets from coughing, sneezing, or direct contact. 27 The fecal–oral route may also be a potential mode of SARS-CoV-2 transmission; however, it has not yet been identified as a vertical route of transmission. 26

### Clinical Presentation and Diagnosis

The clinical manifestations range from mild nonspecific symptoms to severe pneumonia with organ function damage. The common symptoms include fever, cough, fatigue, dyspnea, myalgia, and headache. The possible complications include acute respiratory distress syndrome, shock, acute renal and cardiac injuries, and secondary infections. 27

The initial conclusions in several studies suggested that pregnant or postpartum women did not present a course of aggravated symptoms, and there was no evidence that they were more susceptible to COVID-19 infection. 3 28 However, due to the similarities between SARS-CoV-2 and both Middle East respiratory syndrome coronavirus and SARS-CoV, two other coronaviruses that are known to pose a considerable risk during pregnancy and the puerperium, 25 women in these periods received priority care since the beginning of the pandemic.

Later, cases of maternal deaths associated with COVID-19 raised the question about this matter. 29 In a large international systematic review, it was found that severe cases of COVID-19 were associated with poor maternal and fetal outcomes. 30 To this day, there has been increasing evidence suggesting that COVID-19 can be related to increased need for hospitalization and admission to the intensive care unit (ICU), severe disease, and poor obstetric outcomes in pregnant and in puerperium women. 31 32 33 However, to this day, there are numerous controversial aspects of COVID-19 that remain unexplained, and many of them involve pregnancy, puerperium and childbirth.

The Ministry of Health of Brazil issues, weekly, an epidemiological report including the number of confirmed cases and deaths of pregnant women with COVID-19 and severe acute respiratory syndrome (SARS). Until February 27, 2021, since the beginning of the pandemic, there were 414 maternal deaths associated with SARS, 303 of them with confirmed COVID-19. The same documents appointed 12,027 cases of SARS among pregnant women until February 27, 5,632 of them with confirmed COVID-19. 34 35 Therefore, it is essential to offer priority care to all pregnant and puerperal women with COVID-19 symptoms, and to perform the correct differential diagnosis as quickly as possible.

Typical computed tomography (CT) imaging of the chest shows ground-glass opacity, bilateral patchy shadows, and subsegmental areas of consolidation. An important characteristic of CT manifestations is the remarkable velocity of change. 36

Laboratory findings include lymphopenia, thrombocytopenia, and leukopenia. Many patients show increased levels of D-dimer, which draws attention to the differential diagnosis of TEVs. 37

Real-time reverse transcription polymerase chain reaction (RT-PCR) is the gold standard assay for the laboratory diagnosis of SARS-CoV-2 infection. 38 Chest CT, a highly sensitive diagnostic method for COVID-19, is considered an important tool when RT-PCR is not available. 36

### Prognosis

According to publications, pregnant women and their fetuses represent a high-risk group, especially considering the physiological and mechanical changes that occur during pregnancy. Mothers are vulnerable because of T-helper 2 dominance (which is essential to the protection of fetuses) and cardiorespiratory adaptation. Therefore, a fast diagnosis and prompt treatment by healthcare providers are essential to the prognosis of pregnant women. 28 39 40

## Influenza

Influenza outbreaks occur every year, mainly during the autumn/winter season, infecting and killing hundreds of thousands of people worldwide. 41 Its virulence changes because the virus has a high mutation rate, frequently producing different strains, some of which are responsible for the greatest pandemics in human history. 42 Thus, the severity of influenza can vary depending on the disease subtype. For instance, H5N1 and H7N9 have higher mortality rates than H1N1. 41

Although there is no evidence supporting the hypothesis that pregnancy and the postpartum period, especially the 1 ^st^ 2 weeks after birth, alter the susceptibility of individuals to contracting influenza, women in these periods have a higher risk of severe illness, morbidity, hospitalization, and mortality. 42 43 Another point that should be highlighted is the potential risk of the newborn to contract the infection and its harmful consequences for the infant.

### Clinical Presentation and Diagnosis

The common symptoms of influenza include dyspnea and other typical manifestations of respiratory infections, such as fever, cough, myalgia, headache, fatigue, and arthralgia. The development of complications is not common, except in high-risk groups, including pregnant and postpartum women. These two groups more often present complications such as viral or bacterial pneumonia, pericarditis, myositis, encephalitis, and Guillain–Barre syndrome, which explains their higher ICU admission rates and longer hospitalizations. 44

Although the flu diagnosis can be made clinically, only RT-PCR can confirm the etiology of the virus. 45 Imaging studies, such as chest radiography and CT, do not help in the diagnosis; however, they can be important tools for excluding other possible causes of the symptoms or for identifying conditions that would require an intervention, such as respiratory complications, pneumonia, and pleural effusions. Viral pneumonia shows diffuse, bilateral, patchy interstitial infiltrates, whereas bacterial pneumonia shows focal or unilateral infiltrates. 46

### Prophylaxis and Prognosis

**Chart 1.**
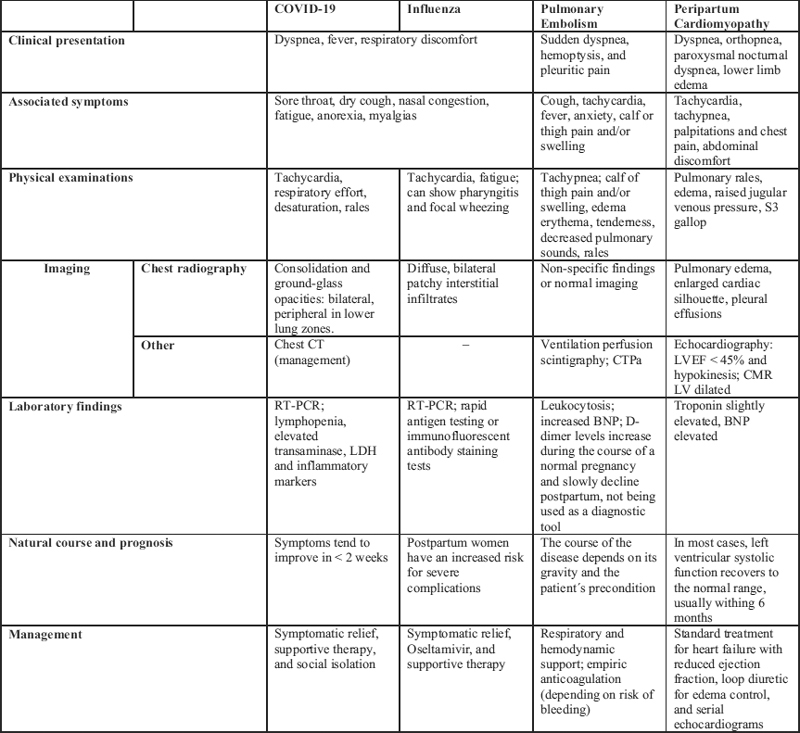
Summarizes the above-mentioned differential diagnoses Abbreviations: BNP, B-type natriuretic peptide; CMR, cardiac magnetic resonance; CTPa, computed tomography pulmonary angiography; LDH, lactate dehydrogenase; LVEF, left ventricular ejection fraction; LV, left ventricle; RT-PCR, reverse transcription polymerase chain reaction.

The best way to avoid influenza infection is through vaccination, which reduces the percentage and severity of these infections, especially in high-risk groups. Since the 2009–2010 H1N1 pandemic, the WHO has defined pregnant and postpartum women as a priority group for vaccination. 44 45 Therefore, it is important for physicians to educate pregnant and postpartum women about vaccination, how to ensure their safety, and their considerable risk of becoming infected, including the risks to their infants ( Chart 1 ).

## Discussion

Dyspnea is a cardinal symptom that is commonly encountered in general practice. A fast and reliable diagnosis ensures the minimization of risks, which may be considerable. 46 However, identifying the underlying cause can be a difficult task, and the relevant conclusion of the present integrative review is that medical history taking remains a cornerstone of the clinical evaluation for dyspnea.

With attention focused on COVID-19 at present, our review aimed to identify important and sometimes fatal conditions that, owing to having some symptomatic similarities with COVID-19, may be neglected or diagnosed late, thus compromising the health of affected patients. Accordingly, an accurate and complete medical history is required to identify the risk factors and onset of symptoms, and to guarantee the necessary care.

Dyspnea is a nonspecific symptom that can be associated with a list of illnesses with a broad spectrum of severity, from the common cold to pulmonary thromboembolism. Therefore, clinicians should be ready to identify all possible underlying conditions. Moreover, dyspnea can be related to a psychiatric disorder, as it appears as the first symptom of some of these diseases (for example, anxiety disorders), although this tends to be neglected by clinicians and patients because of the stigma surrounding these diseases. 47

In studying dyspnea in postpartum women, the higher risk of thrombotic events due to the presence of many predisposing conditions must be mentioned. The Virchow triad, encompassing the three factors of venous stasis, hypercoagulability, and endothelial injury, is the sine qua non of thrombosis. 7 48 These three conditions are met during the postpartum period, especially after cesarean delivery. 49 Therefore, we propose that every woman with dyspnea during the postpartum period should be investigated for thromboembolism, in addition to COVID-19.

Moreover, in the presence of risk factors, such as hypertensive disorders, black ethnicity, obesity, and diabetes, PPCM should be considered an important and sometimes fatal diagnostic possibility. Although rare, prompt and adequate treatment may ensure complete recovery of cardiac function. In this clinical scenario, considering its similarity to other diagnostic entities, a complete and detailed medical assessment provides the only chance to achieve a good outcome. Approximately 800 women die of pregnancy- and childbirth-related complications every day. Most of these deaths can be attributed to delays in the provision of adequate care in health facilities. 50 Hence, prompt access to the possible diagnostic evaluations in cases of dyspnea in a healthcare service is an important step toward the prevention of these deaths.
